# Development and validation of a nomogram model for all-cause mortality risk in patients with chronic heart failure and atrial fibrillation

**DOI:** 10.1186/s12877-024-05059-1

**Published:** 2024-05-29

**Authors:** Lin Guan, Chuan-He Wang, Hao Sun, Zhi-Jun Sun

**Affiliations:** 1 Department of Cardiology, Shengjing Hospital of China Medical University, 39 Huaxiang Road, Tiexi Zone, Shenyang, 110022 China; 2 Department of Clinical Epidemiology and Evidence-Based Medicine, the First Hospital of China Medical University, Shenyang, 110001 China

**Keywords:** Prediction model, Risk assessment, Heart failure, Atrial fibrillation, Chronic disease, Mortality

## Abstract

**Background:**

As the global aging process continues to accelerate, heart failure (HF) has become an important cause of increased morbidity and mortality in elderly patients. Chronic atrial fibrillation (AF) is a major risk factor for HF. Patients with HF combined with AF are more difficult to treat and have a worse prognosis. The aim of this study was to explore the risk factors for 1-year mortality in patients with HF combined with AF and to develop a risk prediction assessment model.

**Methods:**

We recruited hospitalized patients with HF and AF who received standardized care in the Department of Cardiology at Shengjing Hospital of China Medical University from January 2013 to December 2018. The patients were randomly divided into modeling and internal validation groups using a random number generator at a 1:1 ratio. Multivariate Cox regression analysis was used to identify risk factors for all-cause mortality during a one-year follow-up period. Then, a nomogram was constructed based on the weights of each index and validated. Receiver operating characteristic curve, the area under the curve (AUC), decision curve, and calibration curve analyses for survival were used to evaluate the model’s predictive and clinical validities and calibration.

**Results:**

We included 3,406 patients who met the eligibility criteria; 1,703 cases each were included in the modeling and internal validation groups. Eight statistically significant predictors were identified: age, sex, New York Heart Association cardiac function class III or IV, a history of myocardial infarction, and the albumin, triglycerides, N-terminal pro-b-type natriuretic peptide, and blood urea nitrogen levels. The AUCs were 0.793 (95% confidence interval: 0.763–0.823) and 0.794 (95% confidence interval: 0.763–0.823) in the modeling and validation cohorts, respectively.

**Conclusions:**

We present a predictive model for all-cause mortality in patients with coexisting HF and AF comprising eight key factors. This model gives clinicians a simple assessment tool that may improve the clinical management of these patients.

## Background

As the population ages and life expectancy increases, the incidence of combined heart failure (HF) and atrial fibrillation (AF) is rising globally. In 30 years, the number of people with AF in the Asian population is estimated to exceed 72 million, and approximately 3 million will have a stroke [1]. Furthermore, approximately 64.3 million people worldwide have chronic HF [2, 3]. As a result, efforts are being made to reduce the burden on society and global healthcare expenditures. As the “two new cardiovascular epidemics of the twenty-first century,” AF and HF are causal and interact closely [4, 5]. The prolonged rapid ventricular rate and irregular rhythm caused by AF can lead to a loss of normal atrial contraction and restricted cardiac filling, eventually leading to heart enlargement and HF. In contrast, the reduced pumping capacity of the heart caused by HF can lead to atrial fibrosis and electrical remodeling, leading to AF and increasing the risk of stroke [6, 7].

Given their co-morbid mechanisms and common risk factors, interventional AF and HF treatments are mutually beneficial. Therefore, developing a prognostic risk prediction model for AF combined with HF is crucial to accurately assess patient prognosis and assist physicians in making targeted clinical decisions. Mortality in patients with AF and HF remains high; thus, clinicians must be more vigilant and regularly assess the risk of adverse outcomes in their patients to minimize the risk of death.

Large-scale studies on predictive models to assess mortality risk in patients with HF combined with AF have not been conducted; only separate AF or HF mortality prediction models exist [8]. Therefore, we established a clinical database by retrospectively collecting admission data on patients with AF combined with HF, constructed a prediction model with the best predictive performance through as few risk factors as possible, and validated and evaluated the model to provide a scientific basis for early detection of high-risk patients and timely interventions by healthcare professionals.

## Methods

### Study population

Clinical data were extracted from the electronic medical records of patients admitted to the Cardiology Department of Shengjing Hospital of China Medical University between January 2013 and December 2018 to establish the retrospective cohort database. Patients diagnosed with HF and AF based on the European Society of Cardiology diagnostic guidelines and those with New York Heart Association (NYHA) cardiac function class II–IV were included [9, 10]. The degree of impaired cardiac function was assessed using the New York Heart Association’s proposed cardiac function grading method, which includes four levels (I–IV). Patients admitted for serious diseases (e.g., severe infection, malignancy, and liver and kidney failure) and those with incomplete data, missed visits, or acute cardiac decompensation due to acute ischemic events, such as acute coronary syndrome (e.g., unstable angina and acute myocardial infarction) were excluded.

The China Medical University Ethics Committee approved this study (approval number: 2019PS594K), which complied with the ethical guidelines for medical research involving human subjects as outlined in the World Medical Association’s Declaration of Helsinki.

### Laboratory indicators, imaging examinations, and detection methods

Peripheral venous blood was taken within 24 h after admission to evaluate the relevant indexes. Biochemical tests, such as liver function, kidney function, uric acid, and serum ions, were performed using Beckman AU5400 or AU5800 biochemical testers (Beckman Coulter, Inc., Carlsbad, CA, United States) Cardiac ultrasound examinations were performed within three days of admission, and the echocardiographic results were used for the analyses. Left ventricular ejection fraction was measured using Simpson’s method. Survival status data were collected from the population death information registration management system of the Liaoning Provincial Center for Disease Control and Prevention. If survival information was unavailable in the database, three-month, six-month, and one-year telephone follow-up data were used instead. All-cause mortality within 12 months was the primary endpoint event. The analysis cutoff time was the date of death.

### Statistical methods

SPSS version 26.0 (IBM Corp., Armonk, NY, USA) was used to establish the database and perform the statistical analyses. All included patients were randomized into groups using a random number generator; 20,230,329 was used as the initial value for the activity generator, and patients with AF and HF were randomized into modeling and internal validation groups using the visualization score box. Normally or approximately normally distributed data were presented as means ± standard deviations to describe the concentration and dispersion trends; between-group differences were assessed using t-test. Medians (P50) and quartiles (P25, P75) were used to describe skewed distribution data; between-group differences were assessed by non-parametric and Mann–Whitney U rank sum tests. The chi-squared test was used as a hypothesis test for the count data, and the number of cases with percentages was used to statistically describe the baseline values. A two-sided test was used for all data. The statistical threshold was usually set at 0.05.

Risk factors for death within one year in patients with HF and AF were screened via multivariable Cox survival regression analysis. R version 4.2.3 (R Core Team, Vienna, Austria) was used to create the nomogram based on the regression coefficients. The model was primarily evaluated based on its discrimination ability, calibration, and clinical validity. Receiver operating characteristic (ROC) curves were plotted, and the areas under the curve (AUCs) were calculated to assess the model’s discrimination ability. The survival calibration curve was plotted to assess the model’s calibration, and a decision curve analysis (DCA) was used to evaluate the clinical validity.

## Results

### General characteristics

We retrospectively collected data on 10,607 patients with HF, of whom 3,406 patients had concomitant AF; 1,703 patients each were assigned to the modeling and validation groups. The modeling group’s mean age was 72 (63, 79) years, and 53% were men. In addition, 66.3%, 19.9%, and 13.8% of patients with HF had preserved, mildly decreased, and reduced ejection fractions, respectively. At the one-year follow-up, there were 210 (12.3%) all-cause deaths. In the validation group, the mean age was 72 (63, 80) years, 52.1% were men, and 65.5%, 20.9%, and 13.9% patients with HF had preserved, mildly decreased, and reduced ejection fractions, respectively. At the one-year follow-up, there were 224 (13.2%) all-cause deaths.

Table 1 presents the baseline characteristics of the study population. Age, sex, NYHA class, a history of myocardial infarction (MI), and the albumin, triglyceride, N-terminal brain natriuretic peptide (NT-ProBNP), blood urea nitrogen (BUN) significantly differed between the survivors and deceased patients in the modeling and validation groups. The deceased patients were older than the survivors, predominantly male, had a poor NYHA classification, and many had diabetes mellitus and a history of MI. Furthermore, the creatinine, BUN, uric acid, NT-ProBNP, cardiac troponin I, and glycosylated hemoglobin levels at admission were higher in the deceased patients than in the surviving patients. In contrast, the hemoglobin, serum sodium, and albumin levels were lower in the deceased patients than in the survivors.

**Table 1:** Comparison of clinical data between the surviving and deceased patients in the modeling and validation groups

Variable	Modeling Group			
	Survive (*n* = 1493)	Mortality (*n* = 210)	Z/x^2^	*P* value
Age(years)	71(62, 79)	77(68, 82)	2.916	0.000
Male, n(%)	777(52.04)	126(60)	4.68	0.031
NYHA Fc			-10.229	0.000
II	624(41.8)	18(8.57)		
III	532(35.63)	90(42.86)		
IV	337(22.57)	102(48.57)		
HR, bpm	90.44(74.00,98.5)	90.44(78.00,108.50)	1.212	0.106
SBP, mmHg	133.77(122,141)	130(115,140)	2.172	0.000
DBP, mmHg	80.66(75, 88)	80.00(70, 81)	2.530	0.000
NT-ProBNP, pg/ml	1856.00 (738.60, 4082.75)	6215.25 (2205.13,12,329.75)	4.976	0.000
cTNI, ng/ml	0.01(0.00,0.05)	0.06(0.03,0.19)	5.261	0.000
Hemoglobin, g/L	132.00(123.00,146.00	128.65(107.83,141.00)	2.432	0.000
BUN,mmol/L	6.85(5.34,8.65)	9.38(6.93,13.87)	4.466	0.000
Creatinine, µmol/L	78.50(64.60,94.30)	100.95(76.45,132.50)	4.410	0.000
UA, µmol/L	427.41(329.90,462.30)	447.00(392.15,628.83)	3.305	0.000
Prealbumin, g/L	0.19(0.16,0.23)	0.16(0.12,0.19)	3.459	0.000
Platelets, 10^∧^9L	180(146,211)	169(130,209.25)	1.491	0.023
Albumin, g/L	38.10(36.10,40.70)	36.30(32.40,38.48)	3.472	0.000
Triglycerides mmol/L	1.06(0.75,1.45)	0.88(0.68,1.24)	1.926	0.001
HDL,mmol/L	0.98(0.80,1.16)	0.92(0.69,1.04)	1.947	0.001
LDL-C, mmol/L	2.43(1.86,2.91)	2.19(1.64,2.69)	1.798	0.003
HbA1c %	6.20(5.70,6.40)	6.37(5.80,6.80)	1.498	0.022
Potassium, mmol/L	4.02(3.75,4.3)	4.08(3.77,4.46)	1.537	0.018
Sodium, mmol/L	140.00(137.90,141.6)	138(135,141)	3.066	0.000
Chlorideion, mmol/L	106.00(103.3,108.2)	103.4(99.85,107)	3.573	0.000
LVEF, %	56(47.13, 61)	47.13(38, 57)	3.579	0.000
LVEDV, ml	134(105, 166)	156(112, 194.25)	2.582	0.000
LVESV, ml	57(42, 83)	79(47, 117.25)	3.164	0.000
**Comorbidities, n (%)**				
CAD, n (%)	852(57.1)	128(61.0)	1.138	0.286
Hypertension, n (%)	920(61.6)	120(57.14)	1.553	0.213
T2DM, n (%)	382(25.6)	71(33.8)	6.377	0.012
Stroke, n (%)	301(20.2)	52(24.8)	2.372	0.124
Previous MI, n (%)	159(10.6)	43(20.5)	17.004	0.000
Current smoker, n (%)	321(21.5)	54(25.7)	1.904	0.168
Alcohol-related, n (%)	230(15.4)	32(15.2)	0.004	0.950
Variable	Validation Group			
	Survive (*n* = 1479)	Mortality (*n* = 224)	Z/x^2^	*P* value
Age(years)	71(62,79)	78(67,83)	3.275	0.000
Male, n(%)	769(52.0)	119(53.1)	0.10	0.752
NYHA Fc			-11.17	0.000
II	634(42.87)	22(9.82)		
III	513(34.69)	82(36.61)		
IV	332(22.45)	120(53.57)		
HR, bpm	90.438(73, 100)	90.438(74.25, 99.5)	0.528	0.943
SBP, mmHg	133.767(122, 142)	130(113, 135)	2.067	0.000
DBP, mmHg	80.655(75, 86)	80(69, 81)	2.270	0.000
NT-ProBNP, pg/ml	1659 (694.7, 3933)	6383.25 (2906.25,12,102.88)	5.838	0.000
cTNI, ng/ml	0.01(0,0.04)	0.04(0.020,0.14)	4.755	0.000
Hemoglobin, g/L	132(122,145)	124.5(106,138)	3.318	0.000
BUN,mmol/L	6.81(5.43,8.41)	9.88(7.25,14.92)	5.122	0.000
Creatinine, µmol/L	77.5(65,92.3)	101.55(80.25,148.38)	4.783	0.000
UA, µmol/L	426.4(326,453)	475.5(416.7,656.8)	4.369	0.000
Prealbumin, g/L	0.19(0.16,0.24)	0.16(0.11,0.19)	3.857	0.000
Platelets, 10^∧^9L	181(150,212)	168(130.25,206)	1.859	0.002
Albumin, g/L	38.1(36,40.6)	36.2(33.2,38.1)	3.731	0.000
Triglycerides mmol/L	1.10(0.77,1.45)	0.985(0.72,1.24)	1.817	0.003
HDL,mmol/L	1.00(0.81,1.18)	0.90(0.69,1.11)	2.247	0.000
LDL-C, mmol/L	2.46(1.91,3.02)	2.27(1.74,2.74)	1.937	0.001
HbA1c %	6.2(5.7,6.4)	6.3(5.8,6.6)	0.790	0.560
Potassium, mmol/L	4.03(3.76,4.26)	4.13(3.77,4.48)	2.036	0.001
Sodium, mmol/L	139.9(137.6,141.5)	138(134.025,141)	3.331	0.000
Chlorideion, mmol/L	105.9(103.3,108.2)	103.55(99.1,107.2)	3.767	0.000
LVEF, %	56.49(47.13,62)	47(40,56.92)	3.827	0.000
LVEDV, ml	134(105,163)	143.5(108,185.75)	2.713	0.000
LVESV, ml	57(42,83)	75.5(47,112)	2.833	0.000
**Comorbidities, n (%)**				
CAD, n (%)	839(56.7)	141(62.9)	3.080	0.079
Hypertension, n (%)	879(59.4)	110(49.1)	8.517	0.004
T2DM, n (%)	386(26.1)	67(29.9)	1.448	0.229
Stroke, n (%)	292(19.7)	63(28.1)	8.283	0.004
Previous MI, n (%)	157(10.6)	46(20.5)	18.235	0.000
Current smoker, n (%)	312(21.1)	53(23.7)	0.760	0.383
Alcohol-related, n (%)	248(16.8)	33(14.7)	0.585	0.444

Values are presented as mean ± SD, median (IQR), or n (%) as appropriate

*Abbreviations*: *NYHA* New York Heart Association, *HR* Heart rate, *SBP* Systolic blood pressure, *DBP* Diastolic blood pressure, *NT-proBNP* N-terminal pro-B-type natriuretic peptide, *cTNI* Cardiac troponin I, *BUN* Blood urea nitrogen, *UA* Uric Acid, *HDL-C* High-density lipoprotein cholesterol, *LDL-C* Low-density lipoprotein cholesterol, *HbA1c* Glycosylated Hemoglobin, *LVEF* Left ventricular ejection fraction, *LVEDV* Left ventricular end-diastolic volume, *LVESV* Left ventricular end-systolic volume, *CAD* Coronary artery disease, *T2DM* Type 2 dibetes mellitus

### All-cause mortality risk factors

Statistically significant variables from the univariate Cox regression analyses were analyzed via multivariable regression analysis. Age ≥ 65 years, male sex, an albumin level of < 30 g/L, a triglyceride level of < 1.7 mmol/L, an NT-ProBNP level of > 5000 pg/mL, a BUN level of > 9.2 mmol/L, NYHA class III or IV, and a history of MI were risk factors for poor prognosis after one year (all *P* < 0.05; Table 2).

**Table 2:** Results of multifactorial and univariate Cox regression analysis of factors influencing the occurrence of adverse outcomes in patients with AF combined with HF

Variable	Univariate analysis			Multivariate analysis		
	***P***	***HR***	***95%CI***	***P***	***HR***	***95%CI***
Age(years)	0.000	2.090	1.460–2.992	0.004	1.737	1.195–2.525
Male, n(%)	0.032	1.353	1.027–1.783	0.021	1.416	1.053–1.904
NYHAFc(III)	0.000	5.457	3.290–9.052	0.000	3.160	1.866–5.350
NYHAFc(IV)	0.000	9.398	5.693–15.512	0.000	4.626	2.701–7.923
Heart rate, bpm	0.015	1.438	1.072–1.929			
NT-ProBNP	0.000	4.288	3.268–5.626	0.000	1.844	1.362–2.497
Anemia	0.000	2.540	1.891–3.412	0.052	1.367	0.997–1.874
BUN	0.000	3.687	2.812–4.833	0.000	1.747	1.283–2.379
Hyperuricemia	0.000	1.812	1.368–2.401	0.382	1.150	0.841–1.574
Platelets	0.051	1.716	0.998–2.951			
Hypoproteinemia	0.000	3.463	2.184–5.491	0.030	1.706	1.052–2.766
Hyperlipemia	0.000	0.358	0.212–0.606	0.008	0.484	0.283–0.829
HbA1c %	0.058	1.310	0.991–1.732			
Hypokalemia	0.056	1.475	0.991–2.195			
Sodium	0.000	2.887	2.092–3.985			
**Comorbidities, n (%)**						
CAD	0.268	1.170	0.887–1.543			
Hypertension	0.205	0.838	0.637–1.101			
T2DM	0.011	1.452	1.091–1.933	0.066	1.313	0.982–1.755
Stroke	0.126	1.277	0.933–1.747			
Previous MI	0.000	2.031	1.452–2.839	0.031	1.456	1.036–2.048
Current smoker	0.165	1.245	0.914–1.697			
Alcohol-related	0.952	0.988	0.678–1.440			

*Abbreviations*: *NYHA* New York Heart Association, *BUN* Blood urea nitrogen, *HbA1c* Glycosylated Hemoglobin, *CAD* Coronary artery disease, *T2DM* Type2dibetes mellitus

According to the ROC curve analysis, compared with age or albumin (or other predictors) alone, the nomogram model constructed in this study containing 8 independent risk factors had the highest diagnostic efficacy, with an AUC of 0.793 (95% CI: 0.763–0.823), which was superior to the other 4 single models (*P* < 0.01), of which the AUC of the albumin prediction model was 0.6812 (0.6395–0.7229), BUN: 0.7051 (0.6665–0.7438), NT-probnp: 0.7397 (0.7041–0.7752), age: 0.6280 (0.5879–0.6681).

### Nomogram mortality risk prediction model

**Fig. 1:**
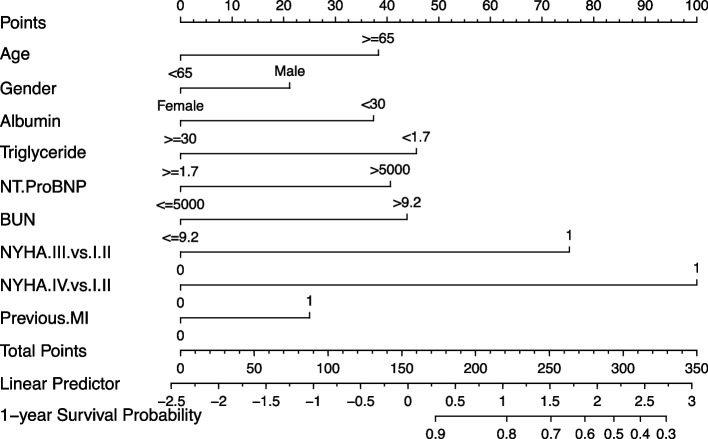
The nomogram model for predicting the one-year mortality risk in patients with HF and AF

The nomogram model for predicting the one-year mortality risk in patients with HF and AF was created based on the multivariate Cox regression analysis using R software (Fig. 1). The left endpoints of each score of the nomogram model corresponded to 0, and the right endpoints were 38, 21, 37.5, 46, 41, 44, 75, 100, and 25 points, representing the number of points for each indicator (Table 3). As the score increases, the risk of death within one year in this patient population increases.

**Table 3:** The scoring table of the prediction model

Risk factors of mortality	Category	Point
Age	< 65 Years	0
	≥ 65 Years	38
Sex	Female	0
	Male	21
Albumin	≥ 30	0
	< 30	37.5
Triglyceride	< 1.7	46
	≥ 1.7	0
NT-ProBNP	≤ 5000	0
	> 5000	41
BUN	≤ 9.2	0
	> 9.2	44
NYHA	NYHAclass I/II	0
	NYHAclass III	75
	NYHAclass IV	100
Previous MI	No	0
	Yes	25

*Abbreviations*: *NT-proBNP* N-terminal pro-B-type natriuretic peptide, *BUN* Blood urea nitrogen, *NYHA* New York Heart Association, *MI* Myocardial infarction

### Nomogram instructions

Per patient:

1. Calculate each risk factor’s score (row 1).
2. Add the scores for all risk factors to calculate the total score (third to last row).
3. The value corresponding to the total score vertically downward is the probability of an all-cause mortality event within one year (last row). 

For example, a male patient (21 points) with HF and AF aged 65 years (38 points) and hypoalbuminemia (37.5 points), NYHA class III (75 points), an NT-ProBNP level of 7000 pg/mL (41 points), a BUN level of 9.5 mmol/L (44 points), and a history of MI (25 points) has a total score of 281.5 points, corresponding to a risk score of 0.65. This score indicates that this patient has a 65% chance of dying within one year and, thus, is a high-risk patient. Table 2 presents the prediction model’s scoring table.

### Clinical validity of the model

**Fig. 2:**
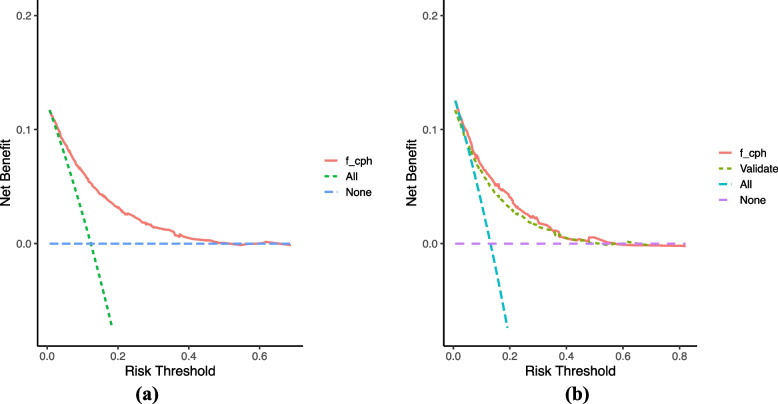
**a** Decision curve analysis (DCA) of the nomogram for predicting the risk of one-year mortality in patients with HF and AF in the modeling group. **b** DCA of the nomogram for predicting the risk of one-year mortality in patients with HF and AF in the validation group. Note: The decision curve’s horizontal coordinate is the threshold probability, and the vertical coordinate is the net benefit. The horizontal line (none) indicates that the net benefit is 0 assuming no all-cause deaths occur in all participants. The left sloping line (all) indicates the net benefit decreases as the threshold probability increases, assuming all-cause deaths occur in all participants. The model has a net clinical benefit when the model’s decision curve is in the upper right of the two extreme lines

Figure 2 presents the DCA results for the nomogram. The modeling (Fig. 2A) and validation (Fig. 2B) sets had a net clinical benefit. Therefore, except for a small range of low preferences, selective patient intervention based on the predictive model has a higher benefit than intervention with all patients and no intervention, suggesting that interventions based on the model will improve clinical outcomes.

### Calibration degree

**Fig. 3:**
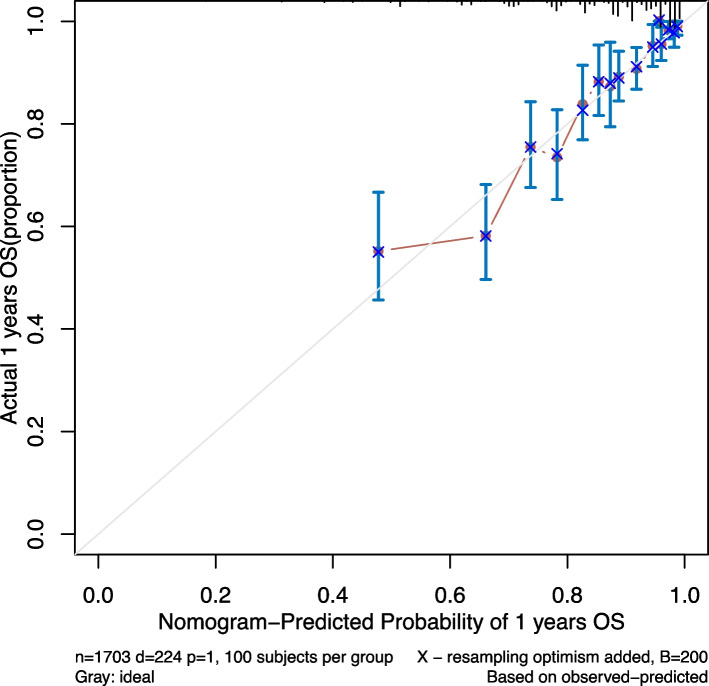
Calibration curve of the nomogram for predicting the risk of one-year mortality in patients with HF and AF

The prediction model for mortality events had good agreement with the predicted risk and the actual occurrence risk in the modeling and validation groups. Figure 3 shows the prediction model’s calibration curve.

### Predictive efficacy

**Fig. 4:**
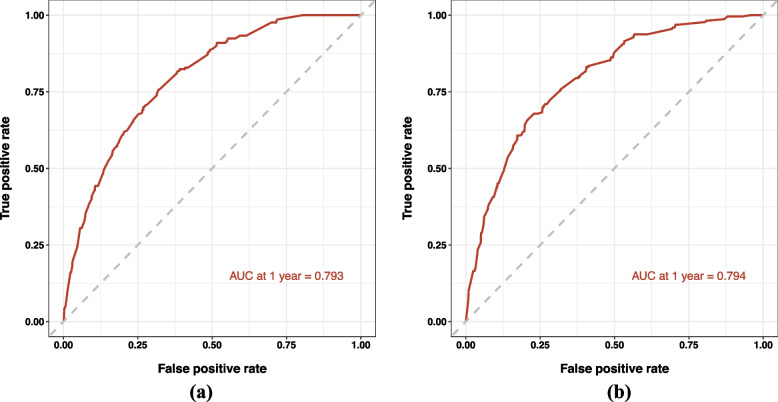
**a** Receiver operating characteristic curve (ROC) for predicting mortality within one year in patients with coexisting AF and HF in the modeling group; **b** Receiver operating characteristic curve (ROC) for predicting mortality within one year in patients with coexisting AF and HF in the validation group

In the modeling group, the AUC was 0.793 (95% confidence interval: 0.763–0.823) for predicting mortality within one year in patients with coexisting AF and HF (Fig. 4a). The AUC was 0.794 (95% confidence interval: 0.763–0.823) in the validation cohort (Fig. 4b).

**Fig. 5:**
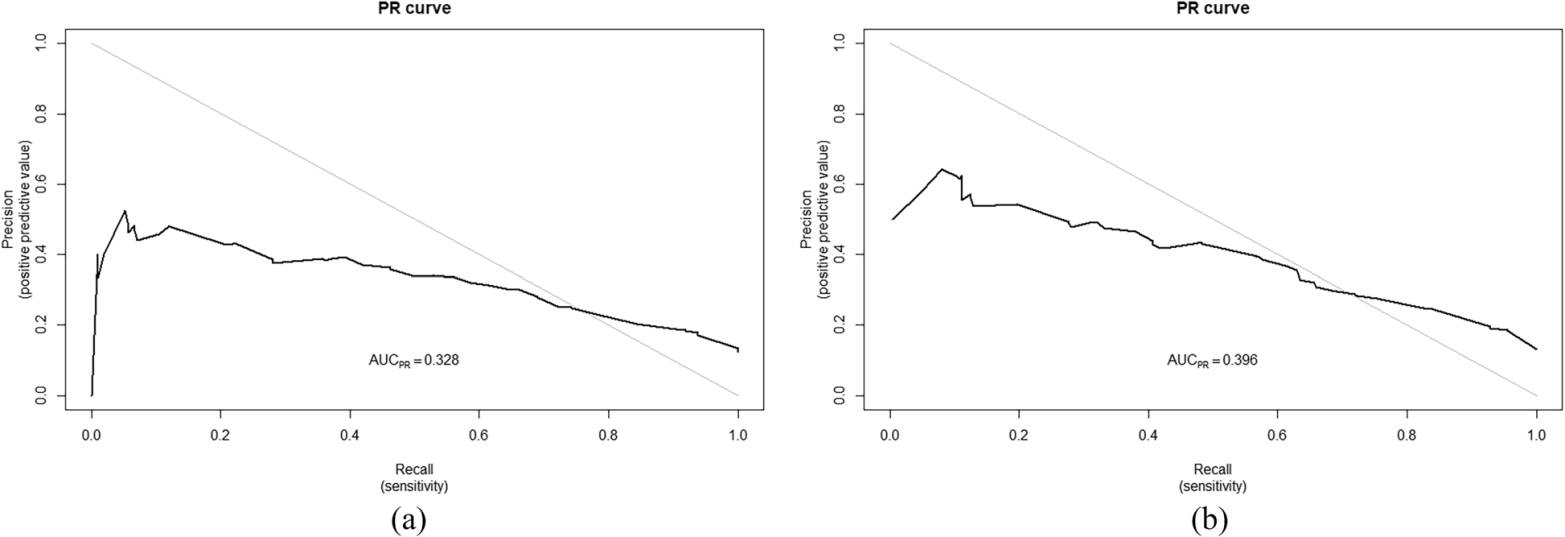
**a** The PR Curve (PRC) for predicting mortality within one year in patients with coexisting AF and HF in the modeling group; **b** The PR Curve (PRC) for predicting mortality within one year in patients with coexisting AF and HF in the validation group

We applied the R language statistical analysis software to plot the PR curves of the predictive model. From Fig. 5A and B, it can be observed that the area under the PR curve (AUC_PR_) was 0. 328 in the modeling group and 0. 396 in the validation of the mortality prediction model for AF combined with heart failure.

**Fig. 6:**
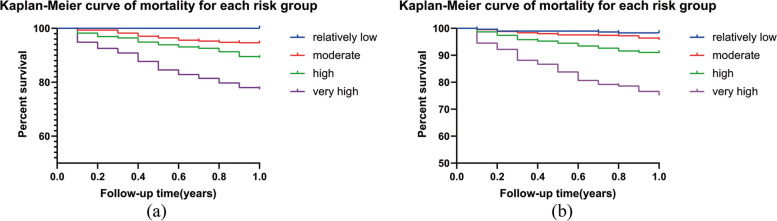
**a** The Kaplan–Meier curves by risk score for the modeling group. **b** The Kaplan–Meier curves by risk score for the validation groups

The 1,703 patients in the modeling group were further divided into four risk-level groups according to quartiles: relatively low (*n* = 285), moderate (*n* = 629), high (*n* = 562), and very high (*n* = 227) risk, corresponding to risk scores < 75, 82–162, 165–245.5, and 246–327.5, respectively. The numbers of patients who died at the end of the 1-year follow-up were 0 (0%), 34 (5.40%), 60(10.68%), and 46(20.26%) in these four groups, respectively. The 1,703 patients in the validation group were divided into four risk-level groups in the same way: relatively low (*n* = 442), moderate (*n* = 523), high (*n* = 603), and very high (*n* = 135) risk, corresponding to risk scores < 87, 90–175, 178–262.5, and 264.5–352.5, respectively. The numbers of patients who died at the end of the 1-year follow-up were 8 (1.81%), 22 (4.21%), 54 (8.95%), and 29 (21.48%) in the four groups, respectively. Figure 6a and b show the Kaplan–Meier curves by risk score for the modeling and validation groups, respectively.

## Discussion

The prevalence of coexisting HF and AF is increasing annually and is concerning because of its high mortality rate and lack of effective treatment. At least one-third of patients with AF also have HF, and at least half of patients with HF also have AF; their coexistence accelerates disease progression and doubles the risk of stroke and death [11]. The CHA2DS2 ~ VASc scoring system for risk stratification of thromboembolism in patients with non-valvular AF is the most widely used clinical antithrombotic scoring system for AF, but few scoring systems assess the mortality risk in these patients. In this study, we developed and validated a mortality risk scoring system that included eight risk factors, providing clinicians with an easy-to-use assessment tool to improve clinical management.

Our results confirm the interaction between these risk factors and death in patients with HF and AF. Other than biological characteristics, such as age and sex, most risk factors can be.

prevented or intervened through lifestyle or diet changes or pharmacological treatment. Thus, raising patients' awareness of relevant risk factors and providing targeted interventions may improve the poor prognostic outcome.

Albumin is the predominant protein in human plasma and an essential nutrient [12, 13]. Evidence supports its protective effect against cardiovascular disease, including AF and HF. Furthermore, many clinical trials have reported that hypoalbuminemia is a strong predictor of increased all-cause mortality [14–16]. Hypoalbuminemia may contribute to patient mortality through antioxidant, anti-inflammatory, and other pathophysiological mechanisms [17, 18]. Therefore, clinical attention should be paid to correcting hypoproteinemia while treating HF. Similarly, our study confirmed hypoalbuminemia as an independent mortality risk factor in patients with HF and AF.

To date, elevated triglycerides have not been documented as a cardiovascular disease-related mortality risk factor, and the relationship between triglycerides and death in patients with HF has not been established [19]. We hypothesize low triglyceride levels are associated with a poor dietary status. A chronic poor nutritional status causes low triglyceride levels and a caloric deficit, resulting in decreased immunity, ultimately increasing the risk of infection and disease. We found that a triglyceride concentration of < 1.7 mmol/L is a risk factor for all-cause mortality in patients with HF and AF.

NT-proBNP is a primary screening test for HF and a biomarker for monitoring the efficacy of HF treatment and assessing prognosis [20, 21]. Specifically, 1,000 ng/L and 5,000 ng/L concentrations are suitable cutoff values for judging long- and short-term acute HF prognoses, respectively [22]. Additionally, the risk of hospitalization and death in patients with chronic HF can be predicted by dynamically monitoring NT-proBNP changes. NT-proBNP is also strongly associated with an increased risk of death in patients with new-onset AF [23]. In our study, 5000 pg/mL was the prognostic cutoff value in patients with HF and AF, and an NT-ProBNP concentration > 5000 pg/mL was a risk factor for all-cause mortality.

In 2022, Yano et al. reported that BUN not only reflects the glomerular filtration rate but also predicts clinical outcomes in patients with HF; they found that elevated BUN levels increase the risk of readmission and all-cause death in patients with HF independent of factors related to renal function [24]. Elevated BUN levels may occur for several reasons, such as excessive consumption of high-protein foods, which increases metabolites and the excreted urea load. BUN levels may also increase from chronic kidney disease, which impairs renal function and decreases the glomerular filtration rate, as well as increased urea reabsorption by the renal tubules. Renin–angiotensin–aldosterone system activation has also been reported to increase urea reabsorption in the proximal tubule, and vasopressin can enhance the reuptake of the collecting duct by activating the urea transport protein, causing an increase in BUN levels [25]. Our study found that a BUN level of > 9.2 mmol/L increased the risk of all-cause mortality by 74.7% in patients with HF and AF.

A prospective observational study from the European Society of Cardiology followed 9,134 patients with HF for up to one year. Their results confirmed the NYHA class as an independent risk factor for prognostic outcome regardless of the ejection fraction category [26]. Our study confirmed that the NYHA class was a prognostic risk factor for those with HF and AF. Compared with NYHA class I and II, the mortality rate was 216% and 326.6% higher in patients with NYHA class III and IV, respectively.

HF is a complication of acute MI, which can cause structural changes in the heart, myocardial fibrosis, and reduced myocardial contractility [27]. Approximately one-fourth of patients who survive MI will develop HF, increasing the risk of long-term mortality [28]. Our study confirmed a history of MI as a risk factor for poor prognosis in patients with HF and AF, which increased the risk of death by 45.6%.

Overall, we found that patients with a score above 24.5 were considered high-risk based on our scoring system, and the one-year mortality rate was as high as 15.3%. Moreover, the eight significant indicators identified in this study have been associated with the prognosis of HF with preserved ejection fraction. Except for age and sex, which are not controllable, intervention for all other factors is possible. For example, healthcare professionals should regularly monitor BUN and NT-proBNP levels and anemia indicators in patients with HF and AF to identify high-risk patients as early as possible and promptly adjust their treatment plan. The latest European Society of Cardiology guidelines suggest that intravenous iron supplementation may prevent adverse outcomes, such as death due to anemia [29], and angiotensin receptor/neprilysin inhibitors reduce NT-proBNP levels and reverse ventricular remodeling [30]; both these interventions may benefit those with HF and AF.

Interestingly, we found that triglyceride may be associated with endpoint events. Lipid metabolism, as an important part of the three major metabolisms of the human body, plays a very important role in the stress state of critically ill patients, as well as sugar and protein metabolism, and is related to the patient's morbidity and mortality. Foreign scholars have concluded that low triglycerides are negatively correlated with the prognosis of patients, and that a reduced or unchanged TC level suggests the progression of infection or organ and metabolic derangement.

This situation can be explained from the point of view of lipid metabolism characteristics.1) Oxidative energy supply: Yang et al. reported that serum free glycerol increased in the death group of critically ill patients compared with the survival group, indicating that at this time, triglycerides were hydrolyzed into free glycerol and free fatty acids under the action of adenylyl cyclase, which provided energy in the early stage of stress, thus decreasing the level of blood triglyceride [31]. 2) Consumption and utilization increased: in patients with severe trauma and sepsis In patients with severe trauma and sepsis, lipoprotein itself binds and neutralizes lipopolysaccharide, resulting in increased lipoprotein consumption and increased cholesterol synthesis and utilization by neoplastic cells.3) Decreased synthesis: In critically ill patients, the decline in plasma proteins and the decline in the hepatic protein synthesis index results in decreased lipoprotein synthesis and decreased serum cholesterol levels because of a decrease in LDL-C. Cholesterol, as a component of cell membrane, is one of the determinants of cell integrity and cell membrane fluidity, and is also an important source of many steroid hormones and certain vitamins, so the decline in lipid levels will inevitably exacerbate the condition, and this lipid metabolism disorders can be causally related to the severity of critical illness. The results of this study show that patients with low triglycerides have a serious condition and a high mortality rate, suggesting that we need to take into account the changes in lipid metabolism in our thinking about saving critical illnesses, which may lead to a more favorable therapeutic outcome.

Less clinical attention has been paid to the risks of combined AF and HF than to the risk of stroke, and therefore attention should be centered on the dangers of AF and HF. Several large-scale clinical studies have confirmed AF and HF-associated risk factors, including age, hypertension, diabetes, and familial hypercholesterolemia [32–36]. However, very few studies have established a risk prediction model to assess the prognosis of patients with coexisting AF and HF. Shuvy et al. analyzed a population of 7,106 patients, reporting that the CHA2DS2-VASc score could be used to assess the risk of thromboembolism and stratify risk in patients with AF, as well as assess prognosis in patients with HF [37]. However, the model included limited clinical indicators and did not include data related to natriuretic peptide levels that are prognostically suggestive, which may have biased the mode’s validity.

Independent serological biomarkers have poor prognostic value in patients with HF and AF. Our combined prediction model based on clinical characteristics and serologic biomarkers had better predictive efficacy than did those that used independent markers. The included predictors are clinically easy to obtain, and this model does not require complex calculations, making it convenient for clinicians. Moreover, the current study internally validated the constructed model in a cohort of 1,703 patients with good results. The risk factor combination had high predictive efficacy in the target population with an AUC of 0.794 (95% confidence interval: 0.763–0.823, *P* < 0.01).

The PR Curve (Precision-Recall Curve) is an important tool to evaluate the performance of a prediction model by considering the True Positive Rate (TPR) and False Positive Rate (FPR) of the model. We applied the R language statistical analysis software to plot the PR curves of the predictive model. From Fig. 5A and 5B, it can be observed that the area under the PR curve (AUCPR) was 0. 328 in the modeling group and 0. 396 in the validation of the mortality prediction model for AF combined with heart failure.The PR curve is a more effective measure of the predictive model when the positive and negative samples are extremely unbalanced, because at this time the upward trend of the ROC curve may be too flat to distinguish between good and bad models.The definition of unbalanced data is negative instances: positive instances > 100:1, the negative instances: positive instances of our data is about 5:1, so it is not unbalanced data [38].

### Limitations

This study has some limitations. First, this is a retrospective clinical study, and the patients in the modeling group are from one center. Therefore, the population characteristics are not universal, and selection bias exists. Second, this study was only internally validated and lacked external validation with data from other centers. Therefore, future large-scale, multicenter, retrospective trials and prospective randomized clinical trials are still needed to support these findings. In the future, as medicine and experimental techniques continue to advance, it is important that researchers look for predictive indicators with stronger correlations to coexisting AF and HF, construct a risk prediction model suitable for patients with AF in China, identify high-risk patients as early as possible, and actively intervene on controllable factors in advance to reduce the occurrence of adverse events.

## Conclusions

We constructed a nomogram with a satisfactory predictive value for one-year mortality in patients with coexisting HF and AF. Except for age and sex, which are uncontrollable, all other risk indicators in our model are clinically treatable. Therefore, we believe that our prediction model will improve the long-term mortality risk assessment and clinical treatment of patients with coexisting HF and AF.

## Data Availability

The datasets used and/or analysed during the current study are available from the corresponding author on reasonable request.

## Abbreviations

NT-proBNP: N-terminal pro-B-type natriuretic peptide
BUN: Blood urea nitrogen
NYHA: New York Heart Association
MI: Myocardial infarction
HR: Heart rate
SBP: Systolic blood pressure
DBP: Diastolic blood pressure
cTNI: Cardiac troponin I
UA: Uric Acid
HDL-C: High-density lipoprotein cholesterol
LDL-C: Low-density lipoprotein cholesterol
HbA1c: Glycosylated Hemoglobin
LVEF: Left ventricular ejection fraction
LVEDV: Left ventricular end-diastolic volume
LVESV: Left ventricular end-systolic volume
CAD: Coronary artery disease
T2DM: Type 2 dibetes mellitus
